# Time-decay Memristive Behavior and diffusive dynamics in one forget process operated by a 3D vertical Pt/Ta_2_O_5−x_/W device

**DOI:** 10.1038/s41598-017-00985-0

**Published:** 2017-04-11

**Authors:** Qi Wang, Deyan He

**Affiliations:** School of Physical Science and Technology, Lanzhou University, Lanzhou, 730000 China

## Abstract

A time-decay resistive switching memory using a 3D vertical Pt/Ta_2_O_5−x_/W device architecture is demonstrated, in which horizontal W electrodes were fabricated, and vertical Pt electrodes was formed at the sidewall after oxide was deposited. Unlike conventional resistive switching, which usually form a conductive filament connect two electrodes, a weak conductive filament was formed from bottom electrode W to near top electrode Pt. The memory can be recovered with a time scale when the electrical stimulation is removed. However, different decay behaviors were observed in one decay curve, including rapid decay and slow decay processes. This can be a good simulation of different stages of forgetting. By a combination of the current decay fitting and the conductive analysis, the rapid decay and slow decay processes correspond to ion diffusion and electron detrapping, respectively.

## Introduction

The ultimate scaling down of the CMOS architecture of modern von-Neumann computer has been plagued by a step known as the von-Neumann bottleneck both with more energy and space consumption. This would require the development of “next-generation” nanodevices have unique functions and characteristics^1^.

Nanoionic devices are some of the most promising candidates for next generation memory and logic devices because of novel characteristics such as their small size, low power consumption and the non-volatility^2–7^. The development of nanoionic devices opened up new applications such as neuromorphic circuits and adaptive system that can mimic efficient artificial neural network in which logic circuits dynamically reconfigure in response to inputs^8–16^. The focus is the implementation of neuroplasticity. In this process, one core is a time decay switch with variable decay time is for the implementation of training process. Among various materials suitable for application in nanoionic devices, Ta_2_O_5_ are promising material for neuromorphic circuits because of their high compatibility with the CMOS fabrication processes.

For Ta_2_O_5_, many reports have connected resistive switching in Pt/TaO_x_ junctions in a negative potential applying to Pt with changes in the oxygen stoichiometry within a thin interfacial dead layer, corresponding to an electron depletion layer caused by the formation of a Schottky barrier. It has showed superior switching speed (sub-nanosecond) and switching endurance (up to 10^12^)^17–20^. The resistive switching in Pt/Ta_2_O_5_ junctions in a forward potential applying to Pt such as widespread reports in Pt/doped-SrTiO_3_^21–23^ was few reports. However, a time-decay resistive switching showed such as in Pt/doped-SrTiO_3_ junctions is much desired for the implementation of neuroplasticity.

In this work, Pt/Ta_2_O_5−x_/W structured devices were investigated in a vertical architecture that is much desired in high density integration^24–30^, which makes the hardware implementation of neuromorphic networks with a comparable number of devices as human’s synapse number possible, and resistive switching properties in Pt/Ta_2_O_5_ junctions in a forward potential applying to Pt was discovered. Unlike conventional resistive switching in other resistive switching devices^31^, the device in this work did not need a forming process and the resistive switching is recoverable. The switching time from the ON state to the OFF state can be controlled by the compliance current. Unconventional decay behaviors were observed in one decay curve, including rapid decay and slow decay processes, when the electrical stimulation is removed in the device. This can be a good simulation of different stages of forgetting. By a combination of the current decay fitting and the conductive analysis, the rapid decay and slow decay processes correspond to ion diffusion and electron detrapping, respectively.

## Results and Discussion

**Figure 1:**
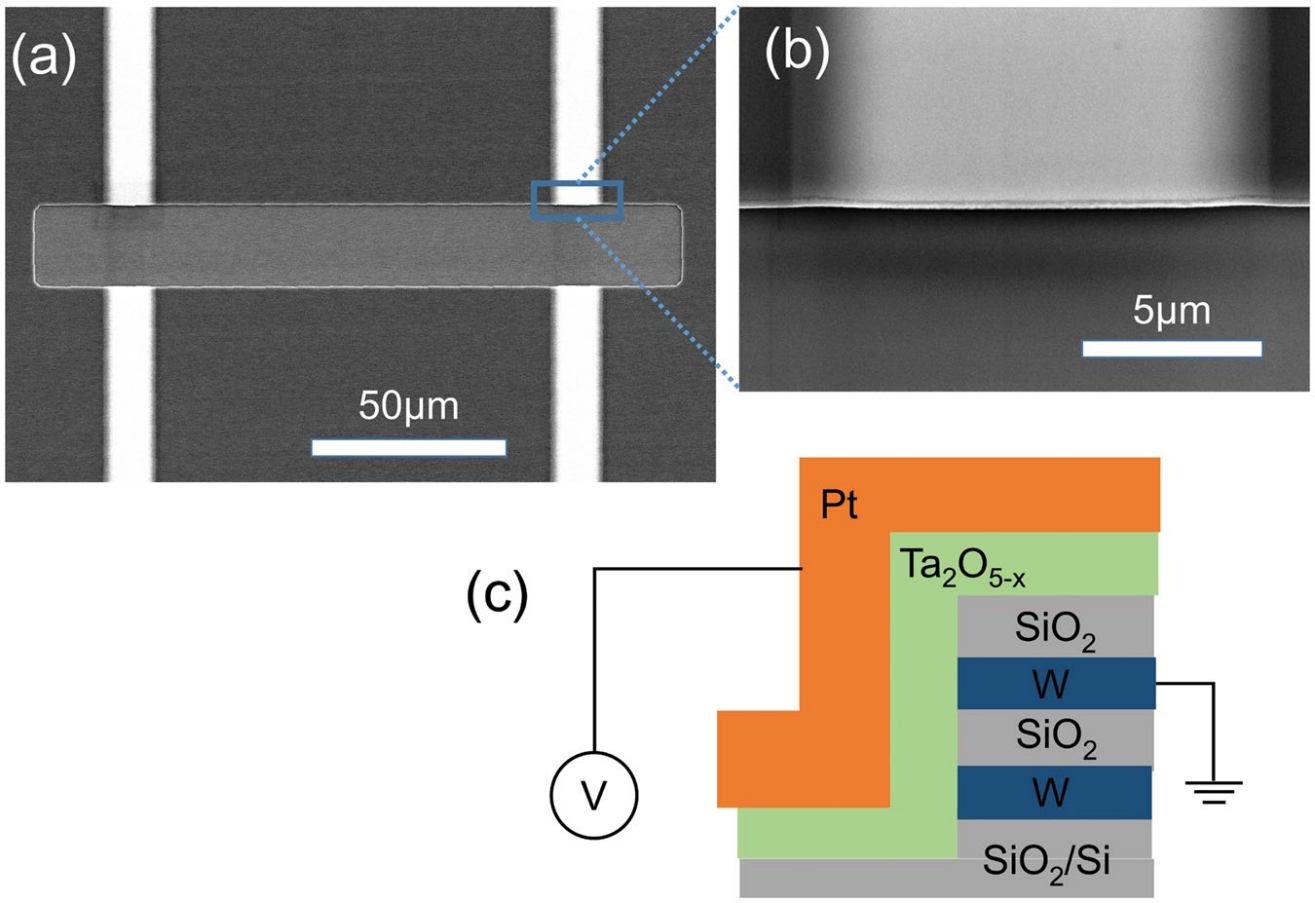
(**a**) Scanning electron microscope image of the 3D W electrodes fabricated by ICP-RIE. (**b**) Scanning electron microscope close-up image of an individual 3D W electrode slide-wall. (**c**) Schematic image of a 3D vertical device Cross-section after fabricating Ta_2_O_5_ and top electrode Pt.

In order to achieve a 3D vertical architecture, we introduced an Inductive Coupled Plasma-Reactive Ion Etching (ICP-RIE) process when fabricating the sidewall of a multi-layer of W, SiO_2_, W, to which Ta_2_O_5_, then Pt was deposited. Figure 1a shows a scanning electron microscope (SEM) image of the 3D vertical W electrodes fabricated by ICP-RIE. Figure 1b shows the close-up image of an individual 3D W electrode slide-wall. The dry-etched region showes a clean interface. Figure 1c shows a schematic image of the 3D vertical device taken after the Ta_2_O_5−x_ layer formation. The W electrodes of the cells were grounded in all electrical measurements. In DC measurements, the sweep speed is 50 mV s^−1^.

**Figure 2:**
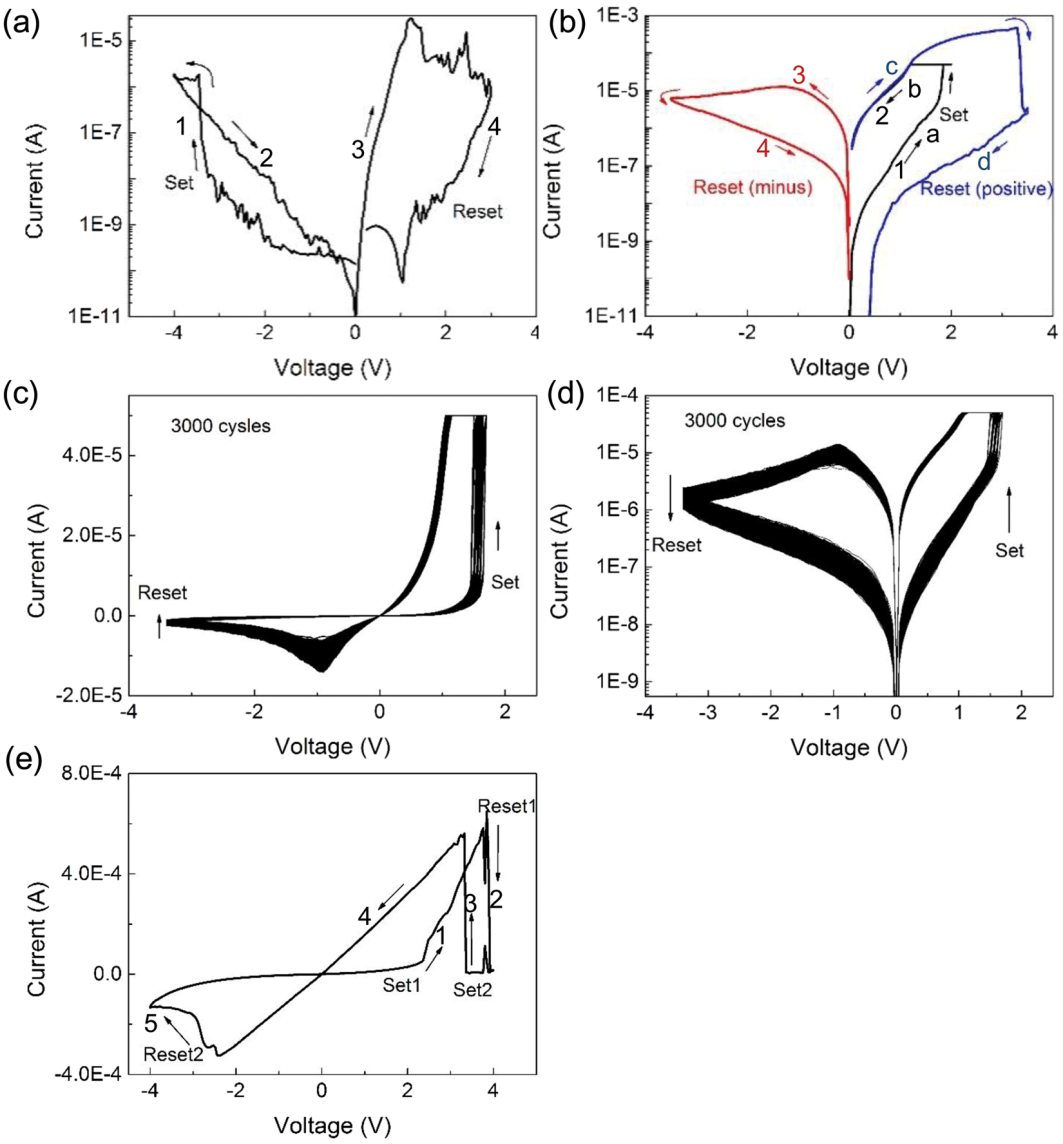
Switching characteristics of a 3D vertical Pt/Ta_2_O_5_/W cell. (**a**) When applying a negative potential to the Pt electrode. (**b**) When applying a positive potential to the Pt electrode, an apparent resistive switching occurs as shown in a black line. Red and green lines separately show the reset I/V curves by a negative and a positive potential. (**c**) 3000 repeated bipolar SET/RESET cycles in a linear coordinate. (**d**) Ditto, current changes are shown as a semi-log coordinate. (**e**) When applying a larger positive potential to the Pt electrode.

The switching characteristics of a 3D Pt/Ta_2_O_5−x_/W cell are shown in Fig. 2. For a pristine cell, when applied a negative potential to the Pt electrode, no apparent resistive switching occurs, as shown in Fig. 2a. After that, the resistance at a large potential could be initiated to a higher resistance state by applying a positive sweep. The devices retained their higher resistance state after a week later at room temperature without any degradation. However, at the initial stage, when applied a positive potential to the Pt electrode, an apparent resistive switching occurs as shown in Fig. 2b. In the measurement, current compliance was set at 50 µA. The voltage for the first set operation is no obvious difference than the subsequent normal set switching, suggesting that no dramatic electroforming is required for the device operation. The voltages were applied on the Pt electrode. Then, a reverse potential reset the cell to high resistance state (HRS). The process is similar to a common bipolar set/reset switch. However, interestingly, after the set operation, the resistance could be triggered to HRS if applying a larger forward sweep potential, as shown in Fig. 2b (blue line). The mechanism should be different from unipolar resistive switch, in which the resistance state is same or smaller than the initial resistance state after reset. The process can be explained as follow,

1 $$T{a}_{2}{O}_{5-X}+xO\to T{a}_{2}{O}_{5}$$

The large forward reset potential caused a growth of the Schottky barriers height and an increase of the depletion layer thickness at the Pt/Ta_2_O_5_ interface by forming non oxygen vacancy doped layer of Ta_2_O_5_. However, a larger forward sweep potential about 4 V will form Ta_2_O_5_ and then destroy the Schottky barrier at the Pt/Ta_2_O_5_ interface, setting the cell to low resistance state (LRS) as shown in Fig. 2e. Figure 2c and d show the repeated bipolar set/reset cycles, where the current compliance was set at 50 μA. In the measurements, on/off ratio was approximately two orders of magnitude. After 3000 cycles, the sweep curves keep no obvious change, suggesting that the cell was not undergoing any irreversible chemical or microstructural changes during the measurements.

**Figure 3:**
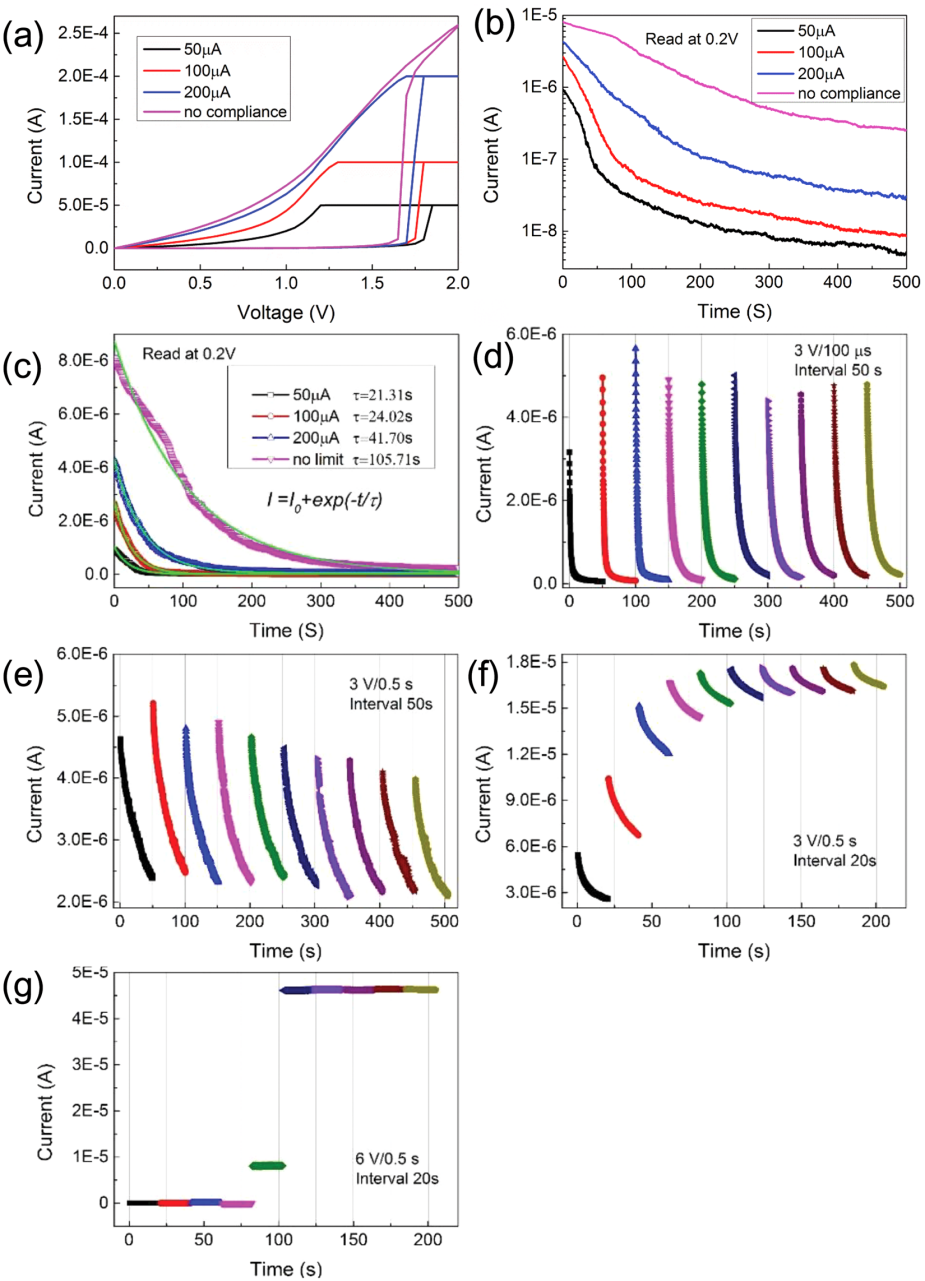
(**a**) I/V characteristics of the Pt/Ta_2_O_5_/W cells. Current compliances were set at 50 µA, 100 µA, 200 µA and no compliance. (**b**) Current read at 0.2 V at room temperature after setting potentials were removed. Changes in current in LRS, as a function of time. (**c**) The data of Fig. 3b in linear coordinate. An exponential function, *I* = *I*_*0*_ + *A exp*(−*t*/*τ*), is used to fit the current decay process after the set with different compliance current. The values of the time constant (τ) extracted from the fitting are plotted in the inset. (**d**) Current changes at room temperature by applying electric pulse of 3 V for 100 µs with an interval of 50 s. (**e**) Ditto, for 0.5 s. (**f**) Ditto, with an interval of 20 s. (**g**) Ditto, by applying electric pulse of 6 V.

The 3D Pt/Ta_2_O_5−x_/W resistance switching cell exhibits memristive behaviors depending on the compliance current conditions, as shown in Fig. 3a. As the compliance current increases, the resistance value at LRS is decreased gradually. Even when the strength of the stimuli are the same, larger compliance current can cause a strong connection maintained persistently. It shows the plasticity of the cell. However, the switching process is volatile, even without compliance current. The current decay process after the set potential with different compliance current in the two-terminal device was systemically investigated. As shown in Fig. 3b, the current read at 0.2 V at room temperature in air was observed to decay acceleratedly at the beginning of 100 s as the compliance current was decreased. At the different compliance current set, current was then observed to use different time to decay back to a same resistance value. A positive correlation exists between the recovery time and the compliance current. Figure 3c shows, *I* = *I*_*0*_ + *A exp*(−*t*/*τ*), is used to fit the current decay process after the set with different compliance current. The values of the time constant (τ) extracted from the fitting are plotted in the inset. The decay time from ON state to OFF state can be controlled by the applied compliance current during the set. This behavior is very similar to that forgetting process that performed by the strength of the stimuli, the number of repetitions of the stimulation and stimulation frequency.

The gradual change of volatile and nonvolatile resistance states are experimentally demonstrated to mimic the human brain’s forgetting process for short-term memory and long-term memory. However, we are more interested in the variation of the resistance states induced by voltage pulse instead of voltage bias. Figure 3d presents the variation of current read at 0.2 V at room temperature in air by applying a sequence of pulse (amplitude 3 v, time 100 us). The high current achieved through inputting forward voltage pulses automatically faded over time but did not return to the original state thoroughly gradually over a measurement period of 50 s after pulse application. After 5 pulses, the decay curves are almost same. That means perhaps the cell decay to a middle state called metaplasticity that suggested by Tan *et al*.^32^. When increased the pulse width to 0.5 s, It is seen the current persisted in a high-level state over a measurement period of 50 s after pulse application. However, the intermediate state, obtained by applying same voltage pulses over a measurement period of 20 s after pulse application, was stable and no temporal fading in the current was observed, as shown in Fig. 3f. In Fig. 3f, the pulse test is under 3 V, less than the critical reset voltage in a forward potential, it is not enough to form Ta_2_O_5_ and decrease the current. But a larger pulse bias as shown in Fig. 3g, different current behavior is obtained over a measurement period of 20 s after pulse application. Small current in the first pulse tests is from forming non oxygen vacancy doped layer of Ta_2_O_5_. However, more pulse tests can destroy the interface barrier. The similar result in a DC sweep is also observed as shown in Fig. 2e. When voltage pulse amplitude increased to 6 v, a strong stimulus with a high frequency triggers the transition from volatile memorization to nonvolatile memorization. This control is analogous to biological synaptic plasticity including short-term plasticity, long-term potentiation, transition from short-term memory to long-term memory, forgetting processes for short- and long-term memory, learning speed, and learning history.

**Figure 4:**
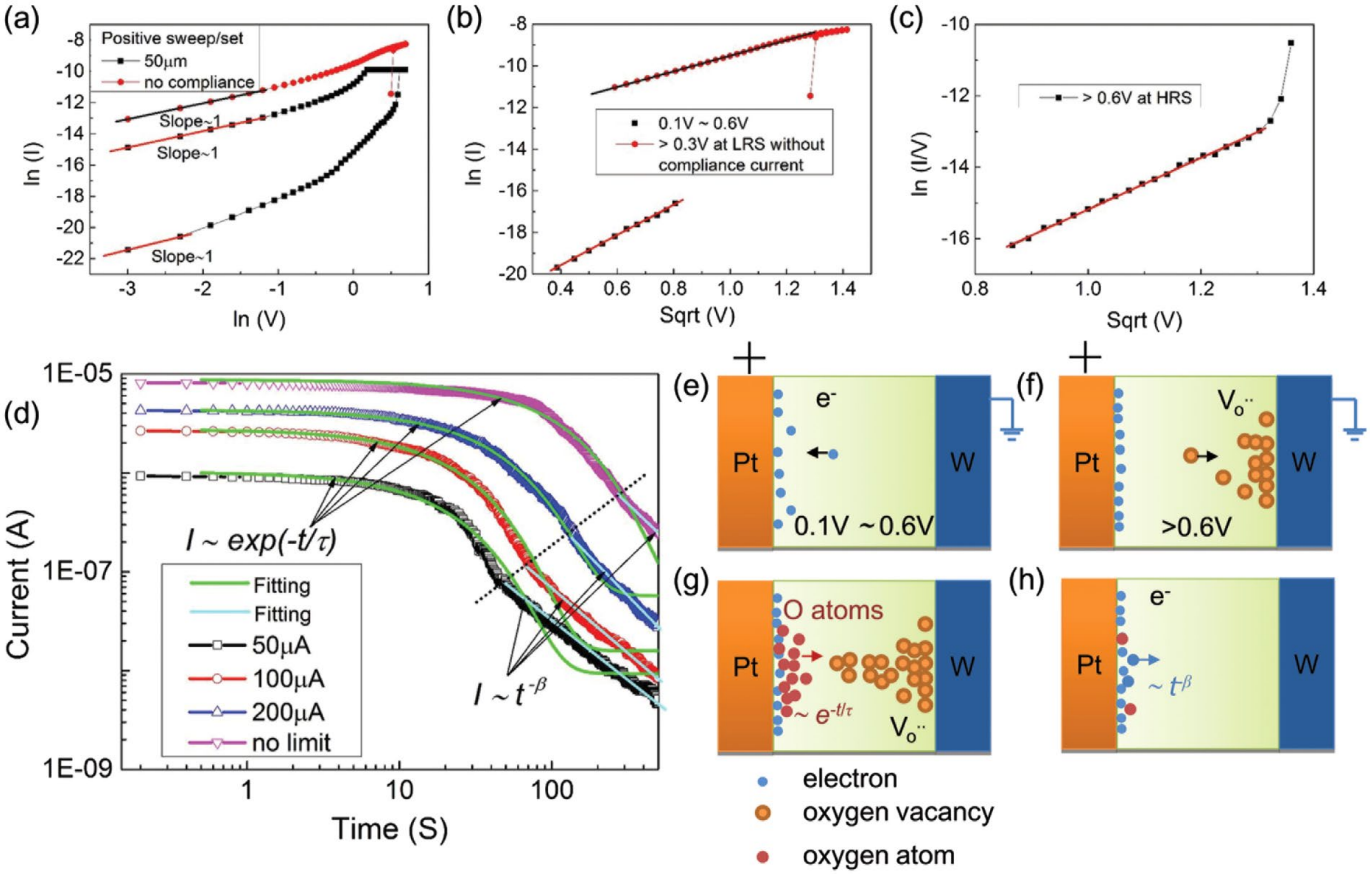
(**a**) Typical I-V curves of set process drawn in double-Ln scale. (**b**) Relation of ln(I) vs. sqrt(V) separately for the forward applied voltage range from 0.1 V to 0.6 V and more than 0.3 V at LRS without compliance current. (**c**) Relation of ln(I/V) vs. sqrt(V) for the forward applied voltage range more than 0.6 V at HRS. (**d**) The current decay processes after set with different compliance current be categorized into two regimes. Before the dash line, Fick’s second law, *I* ~ *exp*(−*t*/*τ*), is used to fit the current decay process. After the dash line, the current decay with time can be well fitted by the Curie-von-Schweidler relaxation law as a *t* ^−*β*^. (**e**,**f**) Schematics of the evolution for conductive channel including electrons and oxygen vacancies by applying a forward set potential. (**g**,**h**) conductive channel decay with time including electrons and oxygen vacancies when removing a forward set potential.

The interesting results inspired us to explore how internal dynamics in cell can affect its conductance. The conducting mechanisms at the LRS and HRS states were analyzed in terms of the current–voltage relation. For a Ta_2_O_5_ dielectric material, by I-V curve fitting, we can preliminarily estimate the memristive switching mechanisms of the device. However, many models of carrier transport mechanism for resistive switching devices have been discussed, including thermionic emission (I/V), Schottky emission (lnI/V^1/2^), Poole-Frenkel emission (ln(I/V)/V^1/2^), Fowler-Nordheim tunneling (ln(I/V^2^)/V^−1^) and trap controlled space charge limited current (I/V^2^). The I-V curves in this paper were replotted in double-Ln scale. The fitting results for set processes are shown in Fig. 4a,b and c. In both of HRS and LRS, the conduction mechanism in the low-voltage region is ohmic behavior due to the slope close to 1, corresponding to the injected excess carriers dominated by the thermally generated carriers and the I-V curve exhibits linear behavior. In HRS, with the applied voltage increasing to more than 0.2 V, the slope is not linear. We attempted to use the different conduction models to fit the I-V curve. It was found that Schottky emission can fit best from 0.1 v to 0.6 v. In addition, asymmetry in the I–V polarities shown in Fig. 2b implies the contribution of Schottky barriers, it can boil down to Schottky emission. Generally, the Schottky emission is the metal/insulator interfacial effect, which arises from lowering of the Schottky barrier under electric field, and can be expressed as follows,

2 $$J={A}^{\ast }{T}^{2}exp[-\frac{q({\varphi }_{B}-\sqrt{qE/4\pi \varepsilon })}{kT}]$$

where J is the current density, A^*^ is the effective Richardson constant, T is the temperature, E is the electric field, *ϕ*_*B*_ is the effective Schottky barrier height, q is the electron charge, ɛ is the dielectric constant, and k is Boltzmann’s constant. Thus, for a given temperature, if we draw the plot of ln(I) versus V^1/2^, a nearly straight line can be obtained. In a forward sweep, to lowering the height of the Schottky barrier, it is hard to explain with the oxygen vacancy migration. The behavior can be explained by considering the trapping and detrapping of electrons within interface states, as proposed in some of the previous reports^33–37^. The origin of interface layer should be from an oxygen vacancy-doped Ta_2_O_5_. Because e-beam evaporation is known to cause more damage from energetic deposition than sputtering, which is why most reports of resistive switching in TaOx based cell are with a reverse potential applying. Under forward bias, the electrons injected from W bottom electrode are trapped by oxygen vacancies in the depletion layer, leading to lowering the barriers height and reducing the depletion layer thickness, as schematically illustrated in Fig. 4e. Therefore, charge trapping/detrapping at the Pt/Ta_2_O_5−x_ interface by forward potential applying modulates the Schottky barrier. When the applied voltage increased further, the corresponding current increased rapidly with a slope of 5. In this case, no gradual current change verse potential (suddenly sharp change) was observed as shown in Fig. 3a, which implied ion migration occurred accompanying the resistance change. Based on a previous report^38^, when Ta_2_O_5−x_ is deposited by EB deposition on a W substrate, it is easy to form an oxygen-deficient layer at the TaOx/W interface. This means the Ta_2_O_5_ layer is more insulating than the W/TaOx interface, so the electric field would drop across the Ta_2_O_5_ film rather than the W/TaOx interface when applying a positive potential, causing oxygen vacancies in Ta_2_O_5−x_ migrate from anode to cathode and the formation a conductive channel from cathode to near anode, as schematically illustrated in Fig. 4f. It was found that Poole-Frenkel emission can fit best, which indicates that Poole-Frenkel emission dominated the charge transport process at high electric field. The equation for Poole-Frenkel emission is given as,

3 $$I=q\mu EA{n}_{0}exp[-\frac{{\phi }_{B}-\sqrt{qE/\pi \varepsilon }}{kT}]$$

where A is the area of the device, *n*_o_ is the defect concentration, and *φ*_*B*_ is the depth of trap from conduction band of Ta_2_O_5_ which is corrected for the electric field in the exponential way, ɛ is the dielectric constant, and k is Boltzmann’s constant. Thus, for a given temperature, if we draw the plot of ln(I/V) versus sqrt(V), a nearly straight line can be obtained, as shown in Fig. 4c. However, in a forward sweep, when the oxygen ions moved to anode interface and reduced to oxygen atom, the oxygen moved along Pt boundaries and was stored in top Pt electrode. After the set, the high concentration of oxygen atoms would diffuse to the conductive channel formed by oxygen vacancies after releasing potential load. So the resistance change is volatile, as observed in Fig. 3b and c. The diffuse process with time meets ~e^−t/τ^, as schematically illustrated in Fig. 4g ^13^. However, it was different to fit the current decay curves with respect to time perfectly as e^-t/τ^ at all time scale. It is even greater apparent observed in a double-Ln scale as shown in Fig. 4d. Significantly, careful analysis of the data shows that the decay appears to occur at very different processes. We exemplify that the current over time characteristics at different compliance current can be categorized into two regimes. After the dash line, the current decay with time can be well fitted by the Curie-von-Schweidler relaxation law as a t^-β^ ^33–36^. The Curie-von-Schweidler behavior is typical for the charge trapping in high-k dielectrics. In light of the above findings, an electron trapping/detrapping effect in interface defect states is postulated because of an oxygen vacancy-doped Ta_2_O_5_ switching layer, as schematically illustrated in Fig. 4h. The electron trapping/detrapping effect widely exists in the metal/oxide heterojunctions. At different compliance current, different decay can be observed through adjusting ion/electron decay ratio. Obviously, both electronic effects such as trapping and detrapping of defect states as well as oxygen-ion migration can well explain the electrical data.

## Conclusions

We demonstrated that the time-decay resistive switching memory with a forward potential to Pt for the first time using a 3D vertical Pt/Ta_2_O_5−x_/W device architecture. The memory can be recovered with a time scale when the electrical stimulation was removed. This recoverable process can emulate the synaptic plasticity including rapid decay and slow decay stages of forgetting in one forget process of human brain. With a combination of the current decay fit and the conductive analysis, both electronic effects such as trapping and detrapping of defect states as well as oxygen-ion migration can well explain the rapid decay and slow decay stages. Beside, 3D vertical device architecture as well as the high compatibility of Ta_2_O_5_ with the CMOS fabrication processes both are much desired for the future brain-like computing system.

## Methods

A Si wafer with a 300 nm SiO_2_ surface covering was chosen as the device substrate. In the first part of the fabrication, the W (30 nm) multiple layer electrodes with 50 μm in width were formed on the substrate by DC sputtering (30 W, 5 mTorr of argon gas) with metal shadow mask, in between which a 5 nm-thick SiO_2_ layer was deposited by RF sputtering (200 W, 5 mTorr of oxygen and argon mixture gas) using an SiO_2_ ceramic target. SiO_2_ (40 nm) was then deposited by the same method as a protective layer. A sidewall of the W electrodes was made by ICP-RIE using an Ar and SF_6_ gas mixture, in which process the non-etched area was covered by a photoresist film. After forming a sidewall by ICP-RIE, 15-nm thick Ta_2_O_5_ layers were formed on the sidewall, by room-temperature electron-beam evaporation. Then, a top electrode of 10 nm Pt was formed on the Ta_2_O_5_ layer by electron-beam evaporation at room temperature through a metal mask. An Agilent 4155C and B1500 semiconductor parameter analyzer were used for electrical characterization. The W electrodes of the cells were grounded in all electrical measurements.
